# Clinical predictors of tocilizumab response in moderate-to-severe thyroid eye disease: a large single-center retrospective cohort study

**DOI:** 10.3389/fendo.2026.1823718

**Published:** 2026-04-27

**Authors:** Ming Lu, Luqiang Wang, Ziqi Wang, Zhihui Song

**Affiliations:** 1 Department of Endocrinology, Beijing Tongren Hospital, Capital Medical University, Beijing, China; 2 Department of Orthopedics, National Cancer Center/National Clinical Research Center for Cancer/Cancer Hospital, Chinese Academy of Medical Sciences and Peking Union Medical College, Beijing, China; 3 Department of Pharmacy, Beijing Tongren Hospital, Capital Medical University, Beijing, China

**Keywords:** interleukin-6, predictive factors, thyroid eye disease, tocilizumab, TRAb

## Abstract

**Background:**

Tocilizumab (TCZ), an interleukin-6 receptor antagonist, is recommended as a second-line therapy for thyroid eye disease (TED), yet the clinical determinants of treatment response remain poorly defined. Identifying reliable predictors of response is essential for patient selection and precision use.

**Methods:**

We conducted a single-center retrospective cohort study of 234 patients with moderate-to-severe TED treated with intravenous TCZ (8 mg/kg every 4 weeks) at Beijing Tongren Hospital from January 2023 to December 2025. Treatment response was defined as a reduction in Clinical Activity Score (CAS) of ≥2 points from baseline. Candidate predictors were entered into a multivariate binary logistic regression. ROC analysis identified optimal cut-off values.

**Results:**

Following TCZ treatment, CAS decreased significantly from 3.42 ± 0.61 to 1.70 ± 0.67 (P < 0.001), and TRAb declined from 10.35 ± 11.99 IU/L to 4.46 ± 6.73 IU/L (P < 0.001). Of 234 patients with complete paired data, 155 (66.2%) were Responders. Responders had higher TRAb (12.17 vs. 7.07 IU/L), fibrinogen (FIB; 2.83 vs. 2.58 g/L), and HDL-cholesterol (HDL-C; 1.37 vs. 1.24 mmol/L), and shorter disease duration (7.91 vs. 10.08 months; all P < 0.05). Multivariate analysis identified four independent predictors: shorter disease duration (OR = 0.962 per month, 95% CI 0.928–0.998, P = 0.038), higher TRAb (OR = 1.036 per IU/L, 95% CI 1.004–1.068, P = 0.025), higher FIB (OR = 1.869 per g/L, 95% CI 1.097–3.184, P = 0.021), and higher HDL-C (OR = 2.665 per mmol/L, 95% CI 1.175–6.041, P = 0.019). The composite model achieved AUC = 0.695 (95% CI 0.625–0.765, P < 0.001).

**Conclusions:**

TCZ produces clinically meaningful and multi-dimensionally validated response with moderate-to-severe TED. Shorter disease duration, higher baseline TRAb, FIB, and HDL-C independently predict response, offering a preliminary framework to inform patient selection. These findings provide the largest real-world evidence to date for guiding precision use of TCZ in TED.

## 1 Introduction

Thyroid eye disease (TED), also known as Graves’ orbitopathy (GO) or thyroid-associated ophthalmopathy (TAO), is an autoimmune orbital inflammatory disorder associated with thyroid dysfunction, primarily Graves’ disease (1, 2). It is the most common cause of orbital disease in adults and remains a cause of visual impairment worldwide (3). Its pathogenesis involves orbital fibroblast activation, excessive hyaluronic acid, adipogenesis, extraocular muscle enlargement, and orbital tissue remodeling. These changes manifest clinically as proptosis, diplopia, and corneal exposure. In severe cases, they may progress to dysthyroid optic neuropathy (DON) (4). These manifestations impair patients’ visual function, appearance, and quality of life, that demands both effective and timely treatment (5).

Intravenous glucocorticoid therapy has been the standard first-line treatment for moderate-to-severe active TED for decades (6). Yet its limitations in clinical practice are well recognized. A proportion of patients show an inadequate or transient response, and disease reactivation after steroid tapering is common. Long-term or repeated use carries the metabolic disruption, infection, and hepatic risks (7). In recent years, alternative immunomodulatory agents have emerged as promising alternatives (8, 9), such as tocilizumab, rituximab and teprotumumab (10, 11).

Tocilizumab (TCZ), a recombinant humanized monoclonal antibody targeting interleukin-6 (IL-6) and blocking IL-6 signaling, was used for active TED (12). IL-6 promotes several processes that sustain TED pathology: fibroblast activation, glycosaminoglycan synthesis, and the differentiation of orbital preadipocytes (13). Serum IL-6 concentrations are elevated in active TED and correlate with clinical activity scores (CAS), providing a mechanistic rationale for targeted blockade (14). Building on its established efficacy in rheumatoid arthritis (15), TCZ’s application in TED has expanded rapidly (16–18). The 2021 EUGOGO guidelines and the 2022 ATA/ETA joint consensus statement now position TCZ as a recommended second-line therapy for patients who are refractory to or ineligible for glucocorticoids (19, 20).

Despite growing clinical interest, the evidence base for TCZ in TED remains limited. Current studies are predominantly small in scale, with most published cohorts comprising fewer than 50 patients (14, 16). Moreover, there is a critical lack of large-scale analyses identifying predictive factors for treatment response, leaving clinicians without a clear framework for patient selection. Most existing data derives from Western populations, and large-scale evidence from Chinese patients remains scarce.

To address these evidence gaps, the present study reports a retrospective cohort of 234 patients with TED treated with TCZ at Beijing Tongren Hospital, one of China’s largest specialized centers for TED management. Our primary objectives were: (1) to evaluate the clinical and serological efficacy of TCZ; (2) to identify the predictors of treatment response through multivariate logistic regression. (3) to assess the safety outcomes including hematological, hepatic, renal, coagulation and lipid parameters.

## 2 Materials and methods

### 2.1 Study design and ethics

This was a single-center retrospective cohort study conducted at the Department of Endocrinology, Beijing Tongren Hospital, Capital Medical University. The study was approved by the ethics committees of our hospital (Approval Number: TREC2025-KY116). Written informed consent was obtained from all patients before recruitment.

### 2.2 Study participants

Patients were recruited at Beijing Tongren Hospital from January 2023 to December 2025 who received intravenous infusion of TCZ for TED were considered for inclusion. Inclusion criteria: (1) Diagnosis of TED established according to the criteria proposed by Bartley and EUGOGO guidelines (19); (2) Moderate-to-severe disease, defined by the presence of at least one of the following: upper eyelid retraction ≥2 mm; moderate or severe soft tissue involvement; proptosis ≥3 mm above normal range for race and sex; constant or inconstant diplopia; (3) Receipt of at least four TCZ infusion during the study period. Exclusion criteria: (1) Active or severe infectious disease, including active tuberculosis; (2) Use of antibiotics or systemic immunosuppressive agents within one month prior to TCZ initiation; (3) Coexisting immune-mediated or hematological disorders; (4) Pregnancy or lactation; (5) Severe hepatic or renal impairment; (6) Malignancy.

### 2.3 Treatment protocol

All patients received TCZ by intravenous infusion at a dose of 8 mg/kg body weight, administered every 4 weeks. The planned treatment course comprised 4 to 6 infusion cycles, individualized according to disease activity, clinical response, and tolerability. Patients with concurrent thyroid dysfunction received concomitant antithyroid drugs (methimazole or propylthiouracil) or levothyroxine, with doses titrated to maintain euthyroidism throughout the treatment period. Laboratory assessments were performed before each infusion cycle. A comprehensive clinical evaluation, including assessment of disease activity, orbital imaging, and serological markers, was conducted at baseline and at a follow-up visit approximately 4 to 6 months after the final TCZ infusion.

### 2.4 Efficacy outcome measures

Disease activity was assessed using the 7-point CAS, evaluated at baseline and at the final follow-up visit, in accordance with the 2021 EUGOGO clinical practice guidelines. CAS was assessed by experienced endocrinologists. The seven items include: spontaneous orbital pain, gaze-evoked orbital pain, eyelid swelling, eyelid erythema, conjunctival redness, chemosis, and caruncle/plica inflammation. Treatment response was defined as a reduction in CAS of ≥2 points from baseline. Thyroid indicators included the thyroid-stimulating hormone (TSH, mIU/L), total triiodothyronine (TT3, nmol/L), total thyroxine (TT4, nmol/L), free triiodothyronine (FT3, pmol/L), free thyroxine (FT4, pmol/L), thyrotropin receptor antibody (TRAb, IU/L).

### 2.5 Safety outcome measures

Safety was evaluated through serial monitoring of laboratory parameters before each infusion cycle. The following parameters were collected: complete blood count including white blood cell count (WBC) and neutrophil count (NET); hepatic function including alanine aminotransferase (ALT) and aspartate aminotransferase (AST); renal function including serum creatinine (Crea); coagulation panel including prothrombin time (PT), PT activity, activated partial thromboplastin time (APTT), fibrinogen (FIB), and D-dimer; and lipid panel including total cholesterol (TC), triglycerides (TG), low-density lipoprotein cholesterol (LDL-C), and high-density lipoprotein cholesterol (HDL-C). Adverse events (AEs) occurring during the treatment period were recorded, including infusion reactions, allergic reactions, infections, and other clinically significant events.

### 2.6 Statistical analysis

All statistical analyses were performed using SPSS software. Normally distributed data are expressed as mean ± standard deviation (SD); non-normally distributed data are expressed as median with interquartile range (IQR). Categorical variables are reported as frequencies and percentages (n, %). Changes in continuous variables from baseline to final follow-up were analyzed using the paired Student’s t-test for normally distributed data, or the Wilcoxon signed-rank test for non-normally distributed data. Univariate analysis. Continuous variables were analyzed using the independent samples t-test or Mann-Whitney U test, categorical variables were analyzed using Pearson’s chi-squared test. Variables with a univariate P-value < 0.10 were considered for inclusion in the multivariate model, entering a binary logistic regression model using the enter method. Discrimination was evaluated by the area under the receiver operating characteristic curve (AUC-ROC) with 95% confidence intervals (CI). Results are reported as odds ratios (OR) with 95% CI and P-values and visualized using a Forest plot. All tests were two-tailed, and statistical significance was defined as P < 0.05.

## 3 Results

### 3.1 Overall treatment efficacy and safety: pre- and post-treatment changes

A total of 234 patients with complete baseline and follow-up data were included in the final efficacy analysis. TCZ treatment produced a significant clinical reduction in CAS across the full cohort. The mean CAS declined from 3.42 ± 0.61 at baseline to 1.70 ± 0.67 at final follow-up (P < 0.001). Serum TRAb declined markedly following treatment. The mean TRAb fell from 10.35 ± 11.99 IU/L to 4.45 ± 6.73 IU/L (P < 0.001). These findings suggested that TCZ exerted the clinical remission of TED and immunomodulatory effect on autoantibody production. FT3 and FT4 were unchanged (P = 0.196 and 0.069), while TSH rose modestly from 2.07 ± 3.23 to 2.60 ± 2.08 mIU/L (P = 0.025), likely reflecting the relatively stable thyroid function during the TCZ treatment. (**Table 1**).

**Table 1:** Pre- and post-treatment changes in clinical and laboratory parameters.

Parameter	Pre-treatment (mean ± SD)	Post-treatment (mean ± SD)	P value
CAS	3.42 ± 0.61	1.70 ± 0.67	<0.001*
TRAb (IU/L)	10.35 ± 11.99	4.45 ± 6.73	<0.001*
FT3 (pmol/L)	5.11 ± 0.99	5.21 ± 0.95	0.196
FT4 (pmol/L)	11.04 ± 3.62	10.51 ± 2.31	0.069
TSH (mIU/L)	2.07 ± 3.23	2.60 ± 2.08	0.025*
WBC (×10^9^/L)	6.19 ± 1.58	5.65 ± 1.64	<0.001*
Neutrophils (×10^9^/L)	3.66 ± 1.32	3.22 ± 1.39	<0.001*
ALT (U/L)	19.76 ± 11.07	24.96 ± 14.28	<0.001*
AST (U/L)	18.92 ± 5.58	22.41 ± 7.98	<0.001*
Creatinine (μmol/L)	64.90 ± 12.74	68.92 ± 13.75	<0.001*
PT (s)	11.42 ± 0.54	11.81 ± 0.71	0.416
APTT (s)	26.60 ± 2.13	25.53 ± 2.57	<0.001*
FIB (g/L)	2.74 ± 0.63	1.89 ± 0.63	<0.001*
D-dimer (mg/L)	0.24 ± 0.23	0.21 ± 0.63	0.474
TC (mmol/L)	4.62 ± 0.96	4.68 ± 0.98	0.344
TG (mmol/L)	1.43 ± 0.98	1.39 ± 0.86	0.508
LDL-C (mmol/L)	2.74 ± 0.83	2.79 ± 0.83	0.316
HDL-C (mmol/L)	1.34 ± 0.39	1.42 ± 0.38	<0.001*

Values are mean ± SD. Paired Student’s t-test. CAS, Clinical Activity Score; TRAb, TSH receptor antibody; WBC, white blood cell count; ALT, alanine aminotransferase; AST, aspartate aminotransferase; PT, prothrombin time; APTT, activated partial thromboplastin time; FIB, fibrinogen; TC, total cholesterol; TG, triglycerides; LDL-C, low-density lipoprotein cholesterol; HDL-C, high-density lipoprotein cholesterol. *P < 0.05.

Both WBC and neutrophil count declined significantly. WBC fell from 6.19 ± 1.58 to 5.65 ± 1.64 ×10^9^/L (P < 0.001), and neutrophils from 3.66 ± 1.32 to 3.22 ± 1.39 ×10^9^/L (P < 0.001), reflecting the known suppressive effect of IL-6 blockade on granulopoiesis. ALT rose from 19.76 ± 11.07 to 24.96 ± 14.28 U/L (P < 0.001) and AST from 18.92 ± 5.58 to 22.41 ± 7.98 U/L (P < 0.001). These elevations were mild. Serum creatinine increased modestly from 64.90 ± 12.74 to 68.92 ± 13.75 μmol/L (P < 0.001), remaining within the normal range in all patients. No clinically significant hepatic or renal impairment requiring treatment modification was recorded. Fibrinogen (FIB) showed the most clinically notable coagulation change, declining from 2.74 ± 0.63 g/L to 1.89 ± 0.63 g/L (P < 0.001). APTT shortened from 26.60 ± 2.13 to 25.53 ± 2.57 s (P < 0.001). PT and D-dimer did not change significantly (P = 0.416 and 0.474 respectively). No spontaneous major hemorrhagic event was recorded. HDL-cholesterol increased from 1.34 ± 0.39 to 1.42 ± 0.38 mmol/L (P < 0.001). Total cholesterol, triglycerides, and LDL-cholesterol did not change significantly (P = 0.344, 0.508, and 0.316 respectively), indicating no adverse lipid effect at the treatment durations studied. (**Table 1**).

### 3.2 Baseline characteristics by treatment response classification

A total of **234 patients** were included in the final efficacy analysis (155 in the responder group, 79 in the non-responder group), yielding an overall response rate of 66.2% (155/234).

The baseline characteristics of both groups are summarized in **Table 2**. The two groups were broadly comparable in terms of age, sex, smoking history, thyroid function parameters, and most laboratory indices. However, several clinically relevant differences were apparent at baseline. Responders had a significantly higher mean CAS (3.53 ± 0.63 vs. 3.22 ± 0.53, P < 0.001), higher serum TRAb (12.17 ± 12.80 vs. 7.07 ± 9.39 IU/L, P = 0.002), higher fibrinogen (2.83 ± 0.69 vs. 2.58 ± 0.48 g/L, P = 0.004), and higher HDL-cholesterol (1.37 ± 0.41 vs. 1.24 ± 0.31 mmol/L, P = 0.015) compared with Non-responders. Responders also had a shorter mean disease duration (7.91 ± 7.47 vs. 10.08 ± 8.20 months, P = 0.045). (**Table 2**).

**Table 2:** Baseline characteristics stratified by treatment response.

Variable	Responders (n=155)	Non-responders (n=79)	P value
Age (years)	50.39 ± 11.20	47.94 ± 12.57	0.129
Female sex, n (%)	99 (63.9%)	49 (62.0%)	0.783
Smoking history, n (%)	40 (26.3%)	22 (27.8%)	0.708
Disease duration (months)	7.91 ± 7.47	10.08 ± 8.20	0.045*
Baseline CAS	3.53 ± 0.63	3.22 ± 0.53	<0.001*
TRAb (IU/L)	12.17 ± 12.80	7.07 ± 9.39	0.002*
FT3 (pmol/L)	5.08 ± 0.94	5.17 ± 1.05	0.464
FT4 (pmol/L)	11.02 ± 3.47	10.90 ± 3.87	0.799
TSH (mIU/L)	2.27 ± 3.74	1.80 ± 2.09	0.315
WBC (×10^9^/L)	6.30 ± 1.59	6.10 ± 1.69	0.382
Neutrophils (×10^9^/L)	3.74 ± 1.34	3.56 ± 1.38	0.353
ALT (U/L)	19.53 ± 11.55	19.98 ± 9.60	0.764
AST (U/L)	18.90 ± 6.69	19.16 ± 4.22	0.751
Creatinine (μmol/L)	65.35 ± 13.20	64.73 ± 12.06	0.731
PT (s)	11.43 ± 0.54	11.44 ± 0.57	0.936
APTT (s)	26.46 ± 2.13	26.75 ± 2.11	0.338
FIB (g/L)	2.83 ± 0.69	2.58 ± 0.48	0.004*
D-dimer (mg/L)	0.24 ± 0.22	0.25 ± 0.27	0.704
TC (mmol/L)	4.65 ± 1.00	4.59 ± 0.88	0.619
TG (mmol/L)	1.40 ± 0.84	1.57 ± 1.27	0.202
LDL-C (mmol/L)	2.79 ± 0.86	2.70 ± 0.76	0.577
HDL-C (mmol/L)	1.37 ± 0.41	1.24 ± 0.31	0.015*

Data are mean ± SD unless stated. Independent-samples t-test (unequal variance where Levene P < 0.05). CAS, Clinical Activity Score; TRAb, TSH receptor antibody; WBC, white blood cell count; ALT, alanine aminotransferase; AST, aspartate aminotransferase; PT, prothrombin time; APTT, activated partial thromboplastin time; FIB, fibrinogen; TC, total cholesterol; TG, triglycerides; LDL-C, low-density lipoprotein cholesterol; HDL-C, high-density lipoprotein cholesterol. *P < 0.05.

### 3.3 Univariate analysis of candidate predictors

Univariate analysis comparing all baseline variables between Responders and Non-responders identified five variables with P < 0.10, qualifying for inclusion in multivariate modeling (**Table 3**). Baseline CAS (P < 0.001) was noted but excluded from the regression model due to its mathematical relationship with the CAS-based response criterion. The remaining four eligible predictors were: disease duration (P = 0.045), baseline TRAb (P = 0.002), baseline FIB (P = 0.004) and baseline HDL-C (P = 0.015). All other variables, including age, sex, smoking, thyroid function indices, hepatic, renal, and lipid parameters, did not reach the threshold for inclusion (all P ≥ 0.10).

**Table 3:** Univariate analysis: baseline variables associated with treatment response.

Variable	Responders (n=155)	Non-responders (n=79)	P value
Baseline CAS	3.53 ± 0.63	3.22 ± 0.53	<0.001
Disease duration (months)	7.91 ± 7.47	10.08 ± 8.20	0.045
TRAb (IU/L)	12.17 ± 12.80	7.07 ± 9.39	0.002
FIB (g/L)	2.83 ± 0.69	2.58 ± 0.48	0.004
HDL-C (mmol/L)	1.37 ± 0.41	1.24 ± 0.31	0.015

Data are mean ± SD. CAS, Clinical Activity Score; TRAb, TSH receptor antibody; FIB, fibrinogen; HDL-C, high-density lipoprotein cholesterol.

### 3.4 Multivariate logistic regression: independent predictors of response

The four eligible variables were entered simultaneously into a binary logistic regression model. The model was statistically significant (χ² = 25.985, df = 4, P < 0.001) with a Nagelkerke R² of 0.149 and satisfactory calibration (Hosmer-Lemeshow test: χ² = 6.036, df = 8, P = 0.643). All four variables were independently associated with treatment response, with complete regression statistics including odds ratios, 95% confidence intervals, and exact P values. (**Table 4**).

**Table 4:** Multivariate binary logistic regression: predictors of TCZ treatment response.

Variable	B	Wald statistic	OR (exp B)	95% CI	P value
Disease duration (months)	−0.038	4.312	0.962	0.928–0.998	0.038
TRAb (IU/L)	0.035	5.042	1.036	1.004–1.068	0.025
FIB (g/L)	0.626	5.3	1.869	1.097–3.184	0.021
HDL-C (mmol/L)	0.98	5.507	2.665	1.175–6.041	0.019
Constant	−2.255	5.429	0.105	–	0.02

OR, odds ratio; CI, confidence interval; TRAb, TSH receptor antibody; FIB, fibrinogen; HDL-C, high-density lipoprotein cholesterol. Enter method. Model fit: χ²=25.985, df=4, P<0.001; Nagelkerke R²=0.149; Hosmer-Lemeshow P = 0.643.

Disease duration was the most statistically robust independent predictor (P = 0.038): each additional month of disease was associated with a 3.8% reduction in the odds of response (OR = 0.962, 95% CI 0.928–0.998). HDL-C had the effect size (P = 0.019): each 1 mmol/L increase was associated with a 2.67-fold increase in response odds (OR = 2.665, 95% CI 1.175–6.041). FIB showed an 87% increase in response odds per 1 g/L increment (OR = 1.869, 95% CI 1.097–3.184, P = 0.021). TRAb showed a 3.6% increase in response odds per 1 IU/L increment (OR = 1.036, 95% CI 1.004–1.068, P = 0.025).

### 3.5 ROC curve analysis and optimal cut-off values

**Figure 1:**
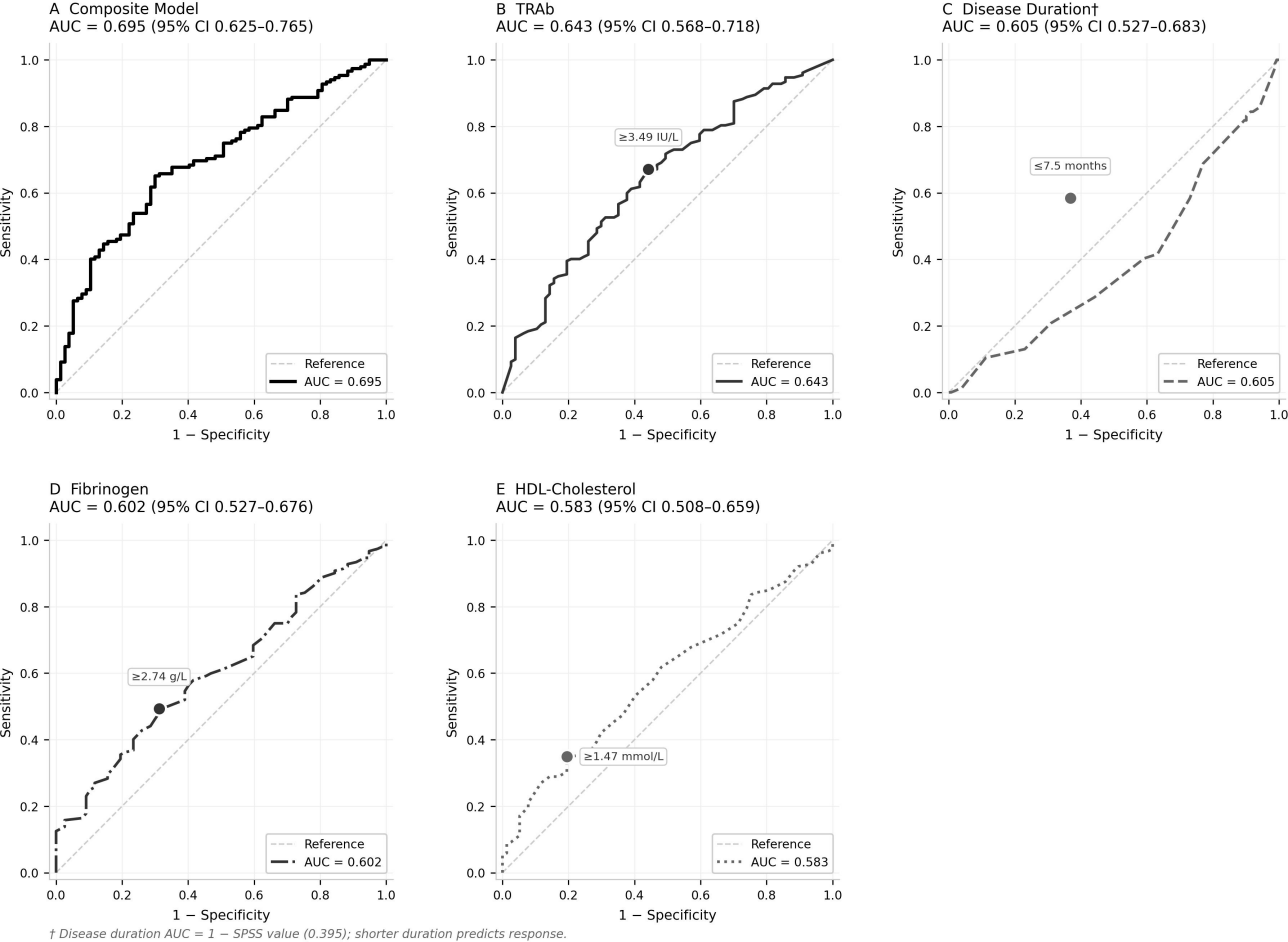
ROC curves for predictors of tocilizumab response. ROC analysis of **(A)** composite model and **(B–E)** individual baseline predictors. Circles mark optimal cut-offs by Youden Index. AUC values and 95% CI shown in panel titles. † Disease duration AUC inverted (1 − 0.395) as shorter duration predicts response.

The composite predictive model achieved an AUC of 0.695 (95% CI 0.625–0.765, P < 0.001), outperforming each individual predictor (**Table 5**; **Figure 1**). Among single predictors, TRAb showed the highest discriminative ability (AUC = 0.643), followed by disease duration after directional correction (AUC = 0.605), FIB (AUC = 0.602), and HDL-C (AUC = 0.583). Optimal cut-off values determined by the Youden Index were disease duration ≤ 7.5 months (sensitivity 58.4%, specificity 63.2%), TRAb ≥ 3.49 IU/L (sensitivity 67.1%, specificity 55.8%), FIB ≥ 2.74 g/L (sensitivity 49.3%, specificity 68.8%), and HDL-C ≥ 1.47 mmol/L (sensitivity 34.9%, specificity 80.5%).

**Table 5:** ROC analysis: AUC and optimal cut-off values for independent predictors.

Predictor	AUC (95% CI)	P	Optimal cut-off	Sensitivity	Specificity
Overall model (PRE_1)	0.695 (0.625–0.765)	<0.001	–	–	–
TRAb baseline (IU/L)	0.643 (0.568–0.718)	<0.001	≥3.49 IU/L	67.10%	55.80%
Disease duration (months)	0.605 (0.527-0.683)†	0.018	≤7.5 months	58.40%	63.20%
FIB baseline (g/L)	0.602(0.527–0.676)	0.013	≥2.74 g/L	49.30%	68.80%
HDL-C baseline (mmol/L)	0.583 (0.509–0.659)	0.037	≥1.47 mmol/L	34.90%	80.50%

†For disease duration, shorter values predict response; AUC reported as 1 − observed value (0.395) to reflect true discriminative direction. Optimal cut-offs by Youden Index (maximum Sensitivity + Specificity − 1).

## 4 Discussion

This study reports the largest single-center, real-world cohort of patients with TED treated with TCZ reported to date. This is the first study to approach TCZ efficacy primarily through the response prediction rather than aggregate outcome reporting. By analyzing 234 cases from Beijing Tongren Hospital, one of China’s largest specialized centers for TED management, our findings provide an evidence-based foundation that extends beyond previous small-scale trials. Our study provides critical real-world evidence for guiding personalized management of TED in clinical practice.

In our study, the overall response rate of 66.2%, is consistent with the previous series, including the landmark study by Pérez-Moreiras et al, Sánchez-Bilbao et al, Farde et al (14, 16, 21). Response rates vary across studies due to differences in patient selection, response criteria, and follow-up duration.

What distinguishes the present findings is the identification of characteristics that independently predicted which patients would respond. Multivariate logistic regression identified shorter disease duration, higher baseline TRAb, higher baseline fibrinogen, and higher baseline HDL-cholesterol as independent predictors of treatment response. This suggested that a simple panel of available clinical and laboratory parameters could meaningfully guide patient selection of TCZ treatment.

Disease duration was the statistically robust predictor in the model (P = 0.038, OR = 0.962 per month), with an optimal cut-off of 7.5 months. In the early phase of TED, orbital inflammation is driven by cytokine-mediated activation of fibroblasts, T-cell infiltration, and glycosaminoglycan deposition, processes in which IL-6 plays a central and well-characterized role (4). As disease progresses, the orbital microenvironment undergoes progressive remodeling: inflammatory cells are gradually replaced by fibroblast-derived extracellular matrix, myofibroblast differentiation occurs, and the tissue transitions toward a fibrotic. In the patient who has longer duration of TED, the proportion of ongoing inflammation that is genuinely IL-6-driven, and therefore amenable to TCZ may be diminished.

This finding has an actionable clinical implication: it provides a basis for the principle of early treatment, which EUGOGO guidelines advocate but do not operationalize with a specific threshold (19). A disease duration cut-off of about 7.5 months may serve as a practical decision aid, prompting clinicians to escalate to TCZ without delay when patients are present with TED. It should be noted that the specificity of this cut-off was modest (63.2%), reflecting the biological heterogeneity of TED and the fact that some patients with longer disease duration still respond. The cut-off should therefore be interpreted as a risk-stratification tool rather than an absolute contraindication.

Baseline TRAb was the predictor with the strongest individual discriminative ability (AUC = 0.643), with an optimal cut-off of 3.49 IU/L. The finding indicated that higher TRAb predicted better response to TCZ. TRAb stimulates orbital fibroblasts largely through downstream cytokine amplification, including upregulation of IL-6 secretion by fibroblasts and infiltrating lymphocytes (12, 22). TCZ’s mechanism of blocking the IL-6 receptor is most effective precisely where IL-6 activity is highest (23). This parallels findings in rheumatoid arthritis, where higher baseline inflammatory markers predict better TCZ response (24). In the broader literature for TED, teprotumumab studies have also identified TRAb as a response predictor (25), suggesting this may be a common feature of biologics targeting inflammatory pathways.

The identification of baseline fibrinogen as an independent predictor of TCZ response (OR = 1.869, P = 0.021) has not been previously reported. Fibrinogen is an acute-phase reactant whose hepatic synthesis is directly upregulated by IL-6 through the JAK/STAT3 pathway, which is the same pathway blocked by TCZ (26). Fibrinogen synthesis is particularly sensitive to IL-6 receptor signaling (27), making it a more specific marker of the very pathway being targeted. Patients with higher baseline fibrinogen have more active systemic IL-6 signaling, suggesting the inflammatory target of TCZ is more prominent. When IL-6 receptors are blocked by TCZ, the fibrinogen-synthesizing axis is disrupted, reflected in reduction in fibrinogen (28), also consistent with our recent Frontiers publication (29).

The finding that higher baseline HDL-C predicted TCZ response (OR = 2.665, P = 0.019, optimal cut-off 1.47 mmol/L) is novel interpretation. HDL-C is conventionally regarded as a cardiovascular risk marker, but accumulating evidence supports a role for HDL particles in immune regulation (30). HDL-C is suppressed during systemic inflammation, insulin resistance, and obesity, which are associated with a high pro-inflammatory IL-6 activity (31–33). In the autoimmune inflammatory disease such as TED, patients with higher HDL-C may enter treatment with an endogenous anti-inflammatory advantage, that is more readily shifted toward resolution when exogenous IL-6 blockade is applied (34). This is speculative but testable, and the inclusion of metabolic parameters in future predictor studies would help resolve this question. Clinically, HDL-C might be useful as predictors, providing additional weight to the decision to initiate TCZ.

The safety profile observed in this cohort is broadly consistent with the established pharmacology of TCZ. Reductions in white blood cell and neutrophil counts, mild transaminase elevation, and modest increases in creatinine reflect the known class effects of IL-6 receptor antagonism and are well-described in both the rheumatology and TED literature (35). Hypofibrinogenemia occurred in 70.9% of the cohort, similar with that reported in most rheumatoid arthritis series (28, 36). We recently published a detailed analysis of TCZ-induced hypofibrinogenemia in this cohort, which provides safety data to the efficacy findings reported here (29).

The moderate discriminative power of our model (AUC = 0.695) underscores the inherent complexity of TED and the limitations of relying solely on clinical parameters. These predictors should be interpreted as risk stratification tools to inform clinical decision-making rather than definitive selection criteria. Future studies may benefit from integrating advanced imaging features through radiomics analysis with broad-spectrum cytokine profiling to develop multi-modal predictive models. Recent work in other immune-mediated diseases has demonstrated the potential of combining clinical, serological, and quantitative imaging biomarkers to improve prognostic accuracy (37, 38). This multi-modal approach may identify distinct inflammatory endotypes within TED that respond differentially to IL-6 blockade.

This study has several limitations. First, the retrospective design introduces inherent selection bias: patients who received TCZ at our center were not randomly allocated, and unmeasured confounders, including referring physician preferences, patient socioeconomic factors, and antithyroid drug adjustment, cannot be excluded. Second, treatment response was defined using a CAS-based criterion, is a subjective composite score susceptible to inter-observer variability. Treatment response was defined solely by CAS reduction, without incorporating measures such as proptosis reduction and diplopia improvement. A composite outcome incorporating multiple dimensions of disease activity, such as orbital imaging data would provide more robust validation. Our future studies should employ multi-dimensional composite endpoints to confirm the clinical utility of these predictors. Third, this is a single-center study from a specialized Chinese TED referral center, and the generalizability of the predictive model to Western populations or community practice settings requires prospective validation. Additionally, our model identified four independent factors, its discriminative power remains moderate (AUC = 0.695). This suggests that clinical and laboratory data alone may not fully capture the biological complexity of TED. Future research integrating radiomics and broad-spectrum cytokine profiling is needed to refine patient stratification and support precision management. The external validation in independent cohorts is needed to confirm the clinical utility of these predictors.

In conclusion, this large real-world cohort study demonstrates that TCZ produces clinically meaningful responses in most patients with moderate-to-severe TED. Treatment response is not uniform: shorter disease duration, higher baseline TRAb, higher baseline fibrinogen, and higher baseline HDL-C each were independently associated with favorable treatment response. These findings provide a preliminary framework for patient selection at the point of TCZ initiation. TCZ is most likely to benefit patients who are present early in the active phase of disease, carry a high autoimmune and inflammatory burden at baseline, and maintain good metabolic health.

## Data Availability

The raw data supporting the conclusions of this article will be made available by the authors, without undue reservation.
